# Advanced searching for hypertrophic cardiomyopathy heritability in real practice tomorrow

**DOI:** 10.3389/fcvm.2023.1236539

**Published:** 2023-07-31

**Authors:** Olga S. Chumakova, Natalia M. Baulina

**Affiliations:** Laboratory of Functional Genomics of Cardiovascular Diseases, National Medical Research Centre of Cardiology Named After E.I. Chazov, Moscow, Russia

**Keywords:** hypertrophic cardiomyopathy, genetics, diagnosis, missing heritability, NGS, HCM-associated variants, polygenic risk score, miRNA

## Abstract

Hypertrophic cardiomyopathy (HCM) is the most common inherited cardiac disease associated with morbidity and mortality at any age. As studies in recent decades have shown, the genetic architecture of HCM is quite complex both in the entire population and in each patient. In the rapidly advancing era of gene therapy, we have to provide a detailed molecular diagnosis to our patients to give them the chance for better and more personalized treatment. In addition to emphasizing the importance of genetic testing in routine practice, this review aims to discuss the possibility to go a step further and create an expanded genetic panel that contains not only variants in core genes but also new candidate genes, including those located in deep intron regions, as well as structural variations. It also highlights the benefits of calculating polygenic risk scores based on a combination of rare and common genetic variants for each patient and of using non-genetic HCM markers, such as microRNAs that can enhance stratification of risk for HCM in unselected populations alongside rare genetic variants and clinical factors. While this review is focusing on HCM, the discussed issues are relevant to other cardiomyopathies.

## 1. Introduction

HCM is the most common inherited cardiac disease affecting at least 1:500 of the general population (1). In adult probands, HCM is defined as the thickening of the left ventricle (LV) wall ≥ (13) 15 mm in the absence of obvious causes for the observed magnitude of hypertrophy (2); this definition includes both familial and sporadic forms of the disease. HCM is characterized by high clinical heterogeneity. Many patients are asymptomatic and early diagnosis is difficult. The others for still unknown reasons develop symptoms of heart failure, atrial fibrillation, and embolic stroke. Risk stratification of sudden cardiac death (SCD), which predominantly affects young people, remains a challenge. The source of such individual clinical differences in HCM is traditionally considered to be its genetic heterogeneity (3).

In the 90s of the last century, the first molecular investigations formed the idea of HCM as a monogenic (Mendelian) “disease of the sarcomere” with autosomal dominant inheritance and age-dependent penetrance (probability that a person with a mutation will develop a disease) with more than 95% affected at 50–60 years of age (4–6). In these cases, the HCM phenotype of the patient is driven by a single rare pathogenic variant of large effect size. Over the next 30 years, new techniques, especially next-generation sequencing (NGS), capable of analyzing large numbers of genes significantly changed our understanding of the genetic architecture of HCM. Importantly, fewer than half of HCM patients who undergo conventional genetic testing show rare pathogenic variants in sarcomeric genes (7). In the sarcomere-negative setting, familial cases are quite common which justifies searching for new causative genes or thinking about a more complex inheritance model based on 2 or more variants of different effect sizes clustering in some families and the impact of shared environment (8). To increase the yield of genetic testing new strategies and techniques (such as whole exome and whole genome sequencing) have been applied, including sequencing a broader range of new candidate genes, screening for copy number variations (CNVs) in known disease-causing genes, and investigation of non-coding regions of DNA. Early disease onset and severe clinical courses often prompt the search for more complex genetic defects such as biallelic or *de novo* mutations (9). Moreover, it is now believed that some HCM patients have non-Mendelian polygenic inheritance patterns triggered by comorbidities (10).

It is also important to consider the common genetic etiology of HCM with other inherited cardiomyopathies. Indeed, the sarcomeric genes play a major role not only in HCM but also in dilated cardiomyopathy (DCM) and restrictive cardiomyopathy (11). Different variants located in different hotspots of the same gene lead to different, sometimes opposite, phenotypes, like HCM and DCM (12). But even the same genetic variant can cause variable clinical phenotypes within the same family that might be related to the disease stage or the impact of other modifiers, so cardiac screening of family members should be aimed at identifying all types of cardiomyopathies.

Over the past decade, other modifiers, which may contribute significantly to the penetrance of mutations associated with HCM, have been explored with some interesting insights from miRNA studies uncovering miRNA potential as prognostic indicators and therapeutic agents along with gene editing. Conducting large-scale studies investigating the miRNA profile to identify crucial HCM-associated miRNAs could increase the accuracy of HCM diagnosis and improve the understanding of the mechanisms of realization of HCM genetic background in the formation of such a variety of clinical phenotypes.

**Figure 1:**
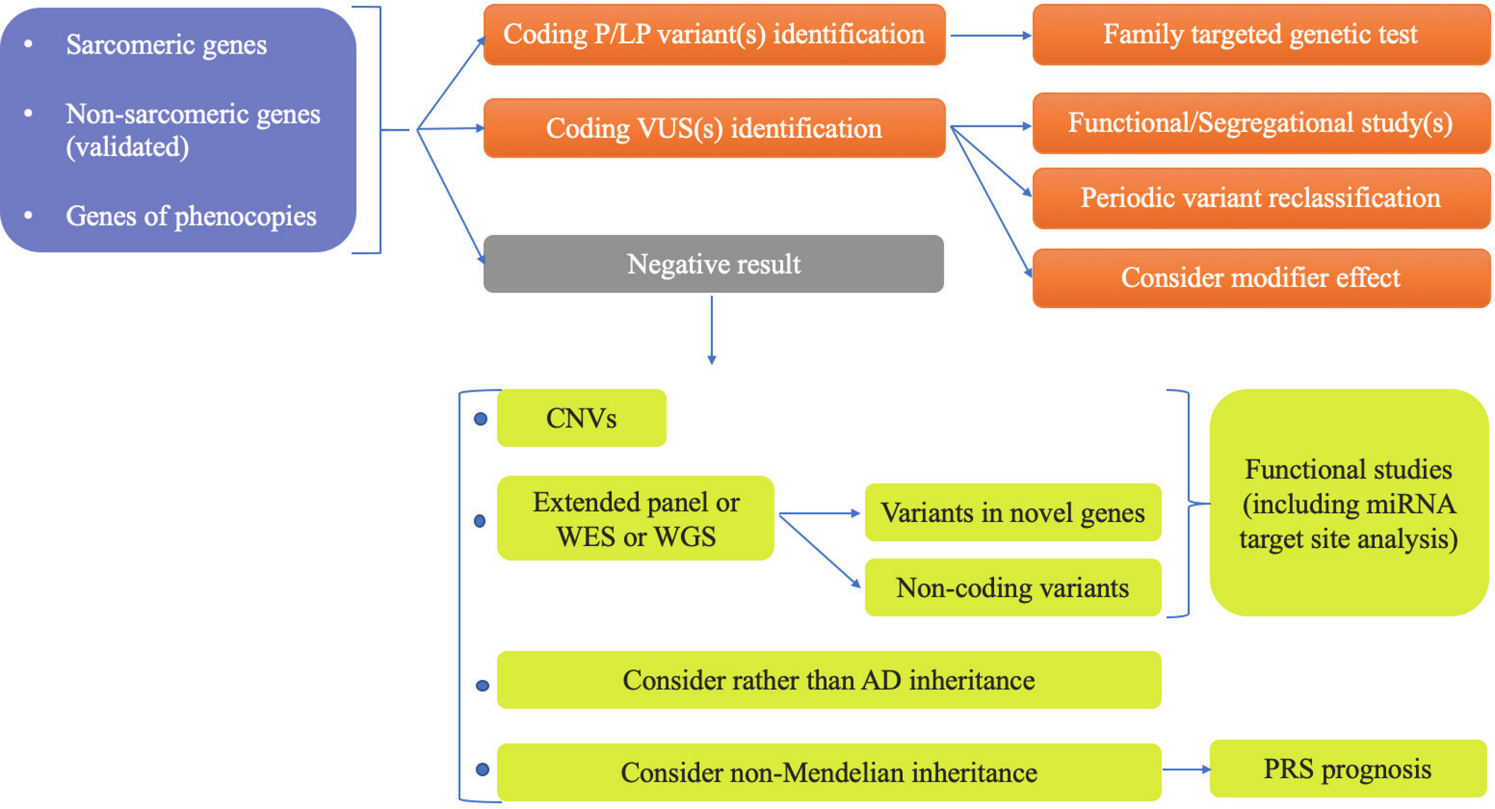
Advanced searching for HCM heritability.

This review summarizes all genetic aspects of current knowledge about HCM and suggests a new advanced genetic panel for patients with HCM phenotype that might be applied if not today, then tomorrow (Figure 1). It also discusses the further directions to supplement this panel with miRNAs that could definitely improve existing imaging-based surveillance protocols to identify and monitor different variant carriers, assess response to treatment, and improve our ability to identify those who may soon progress to clinically overt disease.

## 2. Sarcomeric genes

**Figure 2:**
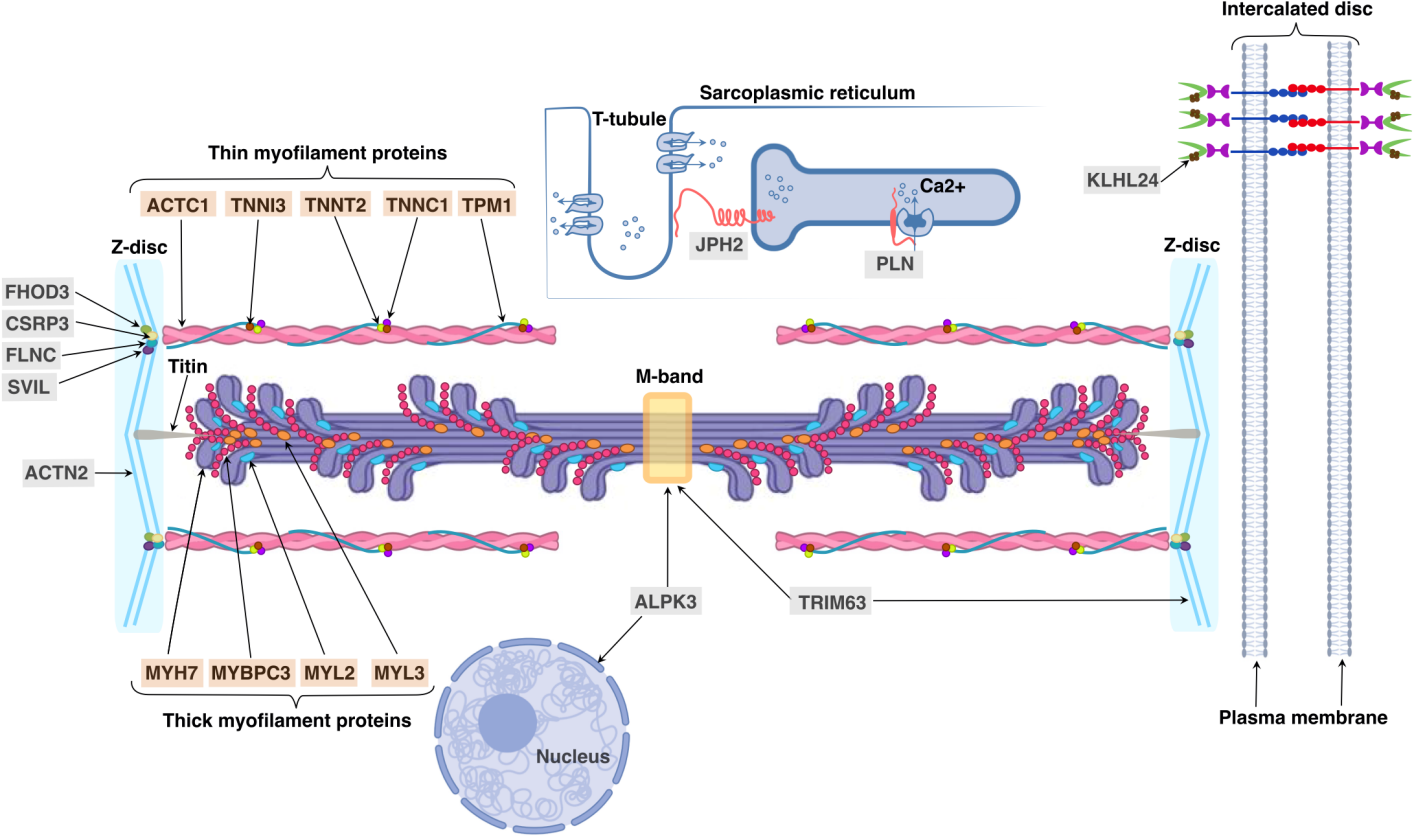
Cellular locations and functions of proteins, encoded by sarcomeric and minor HCM-associated genes. Sarcomeric proteins encoded by HCM-associated genes are indicated with an orange background: ACTC1 (alpha-actin encoded by *ACTC1*), TNNI3 (troponin I encoded by *TNNI3*), TNNT2 (troponin T encoded by *TNNT2*), TNNC1 (troponin C encoded by *TNNC1*), TPM1 (alpha-tropomyosin encoded by *TPM1*), MYH7 (myosin heavy chain 7 encoded by *MYH7*), MYBPC3 (myosin binding protein C encoded by *MYBPC3*), MYL2 and MYL3 (regulatory and essential light chains encoded by *MYL2* and *MYL3*, respectively); minor genes—with a gray background: ACTN2 (alpha actinin 2 encoded by *ACTN2*), ALPK3 (alpha-protein kinase 3 encoded by *ALPK3*), CSRP3 (cysteine and glycine-rich protein 3 encoded by *CSRP3*), FHOD3 (formin homology 2 domain containing 3 protein encoded by *FHOD3*), FLNC (filamin C protein encoded by *FLNC*), JPH2 (junctophilin 2 encoded by *JPH2*), KLHL24 (Kelch-like protein 24 encoded by *KLHL24*), PLN (phospholamban encoded by *PLN*), SVIL (supervillin encoded by *SVIL*) and TRIM63 (muscle-specific RING finger protein 1 encoded by *TRIM63*).

The sarcomere is the contractile unit of cardiomyocytes in which thick filaments (myosin and associated proteins) slide along thin filaments (actin and associated proteins) (Figure 2). Two transverse structures, the Z-disc, and the M-band, anchor thin and thick filaments to the elastic filament system composed of titin. As it has recently been discovered, sarcomere dysfunction manifested as hypercontractility of the heart is a cornerstone of HCM pathogenesis (13). Increased LV contractility is associated with HCM beyond rare sarcomeric variants (14). The effectiveness of a new class of drugs called myosin inhibitors in modulation of LV contractility in HCM patients regardless of their genotype status testifies in favor of the universality of sarcomere dysfunction in HCM development (15) although the difference in the statistical significance of the results between genotype-positive and genotype-negative HCM groups requires further evidence to prove this statement.

Historically, inherited disorders were diagnosed by the phenotype, the segregation of the variant with the disease, or personal and family history. Gene sequencing and the expansion of variants highlighted the necessity of a correct genetic variant classification for managing genetic information. In 2015 the American College of Medical Genetics and Genomics (ACMG) and the Association for Molecular Pathology (AMP) released a universal landmark guidance document for variant classification framework (16). Five-tier classification levels of clinical significance (pathogenicity) of genetic variants have been introduced: pathogenic (P), likely pathogenic (LP), variant of uncertain significance (VUS), likely benign (LB), and benign (B), depending on the applied criteria. According to ACMG guidelines, there are 28 criteria that can be classified by the weight of evidence (strong, moderate, etc.) and type of evidence, i.e., (i) type and location of the variant in the gene; (ii) previously established pathogenicity or, *vice versa*, the novelty or confirmed *de novo* status of variant; (iii) population frequency data; (iv) segregation with patient's phenotype data; (v) evidence of a deleterious effect on the gene or gene product functions based on functional studies or computational (*in silico*) analysis. If there is insufficient evidence to determine disease causality (P/LP) or contrary bystander status (LB/B), the variant is classified as VUS.

Firstly, the genetic basis for HCM has been discovered in 1990 by Seidman's group in collaboration with Prof. William McKenna, who was the most expert in the diverse clinical and echocardiographic manifestations of HCM at that time (17). A point mutation in exon 13 of *MYH7* gene, encoding myosin heavy chain 7, that converts a highly conserved arginine residue (Arg-403) to glutamine in all affected but not in unaffected members of a large kindred riddled by SCD was identified. Clinical examination of over a hundred family members provided clear evidence for the dominant inheritance of this cardiomyopathy and age-related disease expression (5). The compelling evidence of the causative role of *MYH7* in HCM has been received in subsequent studies of unrelated HCM families by identification of other rare non-synonymous variants in *MYH7* gene segregated with HCM (17, 18). However, approximately 50% of families did not reveal mutations in *MYH7* gene; this fact has boosted new genetic linkage studies, which in combination with applying candidate gene approaches resulted in identifying additional disease-causing genes (19). Because the next two recognized HCM-associated genes after *MYH7* were *TPM1* and *TNNT2* encoding contractile proteins alpha-tropomyosin and troponin T, respectively, HCM became a “disease of the sarcomere” (6). The thin filament is composed of alpha-actin, encoded by *ACTC1*, and the calcium-sensitive troponin-tropomyosin apparatus, which includes troponin T, troponin I (*TNNI3*), troponin C (*TNNC1*) and alpha-tropomyosin. Thick filament contains the main molecular motor—cardiac myosin heavy chain 7, regulatory and essential light chains, encoded by *MYL2* and *MYL3* genes, respectively, and myosin binding protein C encoded by *MYBPC3*. Eight aforementioned sarcomeric genes *MYH7* (MIM #192600), *TPM1* (MIM #115196), *TNNT2* (MIM #115195), *ACTC1* (MIM #612098), *TNNI3* (MIM #613690), *MYL2* (MIM #608758), *MYL3* (MIM #608751) and *MYBPC3* (MIM #115197) except *TNNC1* are classified as HCM contributors because they contain some variants with strong evidence of segregation and an aggregate excess of variants in HCM cases compared with controls (20, 21). The *TNNC1* gene has a moderate level of evidence of segregation with HCM because of the relative rarity of its pathogenic variants (MIM #613243) that might be related to the high degree of evolutionary conservation of the gene sequence and/or may be due to severe outcomes at an early age (before reproduction) resulting in the fact that genetic variants are not passed on to new generations (22). About 96% of HCM individuals with positive genetic test results have at least one disease-causative variant in one of eight core sarcomeric genes and two of these genes, *MYBPC3* and *MYH7*, are collectively the most commonly observed (81%) (7).

The age of diagnosis of sarcomeric HCM might be from 0 to >90 years (23, 24) and significant differences in the morphology and clinical course of the disease are reported among the members of one family (25), including monozygous twins (26). Moreover, an extreme difference in penetrance of known sarcomeric mutations is observed between the general population of middle-aged adults (2.6%–6.1%) (27, 28) and the cohort of relatives of genotype-positive HCM probands (up to 50% during follow-up) (29). Such incomplete, age-related penetrance and variable expressivity of P/LP sarcomeric variants may now be explained by polygenic contribution of common variants at a dozen loci (10, 30), particularly those recently found to be associated with a surrogate marker for HCM—cardiac magnetic resonance-derived maximum wall thickness of LV (31). It seems clustering of high-risk common variants-modifiers in the genomes of sarcomeric families would explain the difference in penetrance of the same P/LP mutations between the general population and cohort of relatives, as well as a clinical heterogeneity of HCM within one family (also discussed in “Polygenic HCM” section). The intermediate effect size low-frequency variants also exist in HCM (32). They are assumed to require additional common genetic modifiers for the penetrance (oligogenic inheritance) that make the interpretation of genetic testing results beyond the current capabilities. Harboring such variants in non-sarcomeric genes can offer limited additional sensitivity and some of the disappointment from the expanded genetic panels (33), which resulted in a recommendation of additional screening for only genes associated with HCM mimics, requiring different patient management (34).

A prognostic value of knowledge of whether the patient is harboring a damaging variant or not in one of 8 definitive sarcomeric genes has been convincingly shown in several studies. Compared to sarcomere-negative counterparts, sarcomere-positive patients have a more severe clinical course. They are diagnosed significantly earlier in life, have a worse cardiovascular event-free survival, and higher rate of cardiovascular events (35, 36). The presence of a sarcomeric mutation independently from proband status, sex, and race carried a more than 2-fold increased risk for all HCM-associated outcomes, highest for ventricular arrhythmia, and even after adjustment for earlier age at presentation, hazard remained significantly increased (36). Since a sarcomeric mutation status is predictive of outcomes, the presence of sarcomeric pathogenic variant(s) has recently been incorporated into HCM clinical management guidelines as an additional criterion for implantable cardioverter-defibrillators implantation in patients with intermediate 5-year risk of SCD (37). Unlike sarcomere-positive, sarcomere-negative HCM, after the exclusion of phenocopies and alternative diseases, is significantly less investigated, and its pathogenesis is still unknown. A combination of sarcomere-negative status and negative family history is classified as nonfamilial HCM. This subgroup has demonstrated a more benign clinical course with mortality similar to the age-matched general population, and a much lower familial recurrence risk that should result, according to the authors, in less frequent clinical surveillance of relatives (36, 38). Despite a substantial proportion of sporadic cases, which may have no strong genetic component at all (39), 22% of sarcomere-negative HCM patients have a positive family history (38), which can indicate either an unrecognized Mendelian form of the disease (especially in early-onset or multiple affected relatives), or more complex inheritance model based on variants of different effect sizes clustering in such families (8).

*Sarcomeric genes remain the cornerstone of the genetic panel for HCM screening. In our opinion, most HCM panels comprising only sarcomeric genes must be enriched with additional HCM-associated genetic loci that contribute to the emergence of the individual disease phenotype to improve the efficacy of HCM diagnosis (discussed further)*.

## 3. Non-sarcomeric genes

Despite the vast majority (>90%) of pathogenic variants are located in sarcomeric genes, some HCM-causing variants were found in genes encoding non-sarcomeric proteins. Non-sarcomeric HCM-associated genes can be divided into two groups: “minor” genes harboring mutations that lead to sarcomere dysfunction in a similar manner as mutations located in sarcomeric genes do and genes of phenocopies, or systemic diseases’ genes, with pathogenic variants that may mimic HCM phenotype.

### 3.1. Minor HCM-associated genes

Several comprehensive reviews of less common or “minor” HCM-associated genes with its convincing disease-causing evidence has been recently published (3, 40). Therefore, in this review we accumulate the key data of “minor” HCM genes specifying the location and function of their encoding proteins (Table 1, see Figure 2) as well as their role in HCM development; only the latest data on these genes are discussed below.

**Table 1:** Minor HCM-associated genes.

Gene	Protein	Location of protein	Function of protein	Gene-disease validity classification according to ClinGen (year of approvement)
ACTN2	Alpha actinin 2	Z-disc	Stabilizes sarcomeres by anchors and crosslinks actin filaments from neighboring ones; regulates ion channels	Moderate for AD “intrinsic cardiomyopathy” (2018)
ALPK3	Alpha-protein kinase 3	Between M-band of the sarcomere and nucleus membrane	Maintains sarcomere integrity and proteostasis	Definitive for AR HCM (2022); Not yet evaluated for AD HCM
CSRP3	Cysteine and glycine-rich protein 3 (muscle LIM protein)	Z-disc	Maintains the myocyte cytoskeleton, mechanosensory functions, actin cytoskeleton assembly	Moderate for AD HCM (2017)
FHOD3	Formin Homology 2 Domain Containing 3	Thin filament of sarcomere	Regulates sarcomere organization by polymerization of actin	Not yet evaluated
FLNC	Filamin C	Z-disc	Maintains the integrity of the sarcomere by crosslinking actin filaments at the sarcomeric Z-disc	Not in HCM panel
JPH2	Junctophilin 2	Sarcoplasmic reticulum within junctional membrane complexes	Serves to bridge plasma membrane and sarcolemma, interacts with L-type Ca^2+^ channels, caveolin and RyR2 to regulate Ca^2+^ induced Ca^2+^ release	Moderate for AD HCM (2017)
KLHL24	Kelch-like protein 24	Intermediate filament network	Ubiquitinates different keratins and desmin	Not yet evaluated
PLN	Phospholamban	Calcium signalling	Inhibits cardiac muscle sarcoplasmic reticulum Ca(2+)-ATPase, thereby mediating contraction and relaxation	Definitive for AD “intrinsic cardiomyopathy” (2021)
SVIL	Supervillin	Z-disc	Binds actin and myosin, plays a role in myofibril assembly	Not yet evaluated
TRIM63	Muscle-specific RING-finger protein 1	Z-disc and M-band of sarcomere	Targets cellular proteins, including sarcomeric proteins, for degradation via ubiquitin-proteasome system	Disputed for AD HCM (2022)

AD, autosomal dominant pattern of inheritance; AR, autosomal recessive pattern of inheritance; HCM, hypertrophic cardiomyopathy.

#### 3.1.1. ACTN2

*ACTN2* encodes alpha actinin 2 protein that localizes in Z-disc and is known to stabilize sarcomeres by anchors and crosslinks actin filaments from neighboring ones as well as to regulate ion channels. Variants in this gene has been associated with cardiomyopathies (more often with HCM (MIM #612158) and left ventricular non-compaction cardiomyopathy (MIM #612158)) and skeletal muscle diseases. In 2018 *ACTN2* gene reached a moderate classification of evidence for HCM with a few mutations reported to be segregated in HCM families (41, 42). The list of all known *ACTN2* variants with updated clinical significance, segregation, and/or functional data to support their pathogenicity has been recently published (43). Later, the detailed investigations of the *ACTN2* p.Met228Thr variant in the mouse model identified alpha-actinin 2 protein destabilization as a key disease mechanism. It leads to aberrant activity of the ubiquitin-proteasomal system (UPS) and interferes with cardiomyocyte maturation ensuing mitochondrial dysfunction (44).

#### 3.1.2. ALPK3

*ALPK3* encodes alpha-protein kinase 3 that localizes between M-band of the sarcomere and nucleus membrane and maintains sarcomere integrity and proteostasis. It is thought to be the most rapidly investigated non-sarcomeric gene in association with HCM (MIM #618052). Over 5 years it has been shown that several biallelic and heterozygous variants creating premature stop-codons (truncating variants) in *ALPK3* might be disease-causing (45, 46). The involvement of rare missense *ALPK3* variants in HCM has been recently shown in the Asian population (47). Genome-wide association studies (GWAS) demonstrated the contribution of *ALPK3* common variants in HCM polygenic risk score (PRS) (10, 14). Significant progress in the understanding of the pathogenesis of *ALPK3*-associated cardiomyopathy has been demonstrated in two recent fundamental works. Both study groups came to the same conclusion that (1) alpha-protein kinase 3 localizes to the M-band of the sarcomere and (2) its deficiency dysregulates M-band proteins involved in sarcomere turnover. Thus, *ALPK3* mutations induce hypertrophy by impairment of sarcomere proteostasis (48, 49). Moreover, a protective role of miR-384-5p against cardiac hypertrophy via the alpha-protein kinase 3 signaling pathway regulation has been shown (50).

#### 3.1.3. CSRP3

*CSRP3* encodes cysteine and glycine-rich protein 3 (also known as muscle LIM protein) that localizes in Z-disc and maintains the myocyte cytoskeleton, mechanosensory functions, and actin cytoskeleton assembly. It is a well-established HCM-associated gene despite a small number of reported rare disease-causative variants (MIM #612124). The latest study discovered that *CSRP3* mutations activated UPS-mediated depletion of functional protein, driving the development of non-sarcomeric HCM (51). Moreover, a novel computational screening method for quick identification of key mutation sites for specific protein structures has been recently provided; it was established that the most common *CSRP3* substitution L44P affects the LIM domain structure by altering the secondary structure of the protein (52).

#### 3.1.4. FHOD3

*FHOD3* encodes formin homology 2 domain containing 3 protein that localizes in thin filament of sarcomere. The longest isoform of *FHOD3* is expressed exclusively in the heart and required for sarcomere formation via both thin (actin) (53) and thick (myosin binding protein C) filaments (54). Pathogenic rare *FHOD3* variants associated with HCM are mostly non-truncating and affect the diaphanous inhibitory domain of the protein (HCM, MIM #619402) (55, 56). Role of *FHOD3* variants in DCM is still unclear.

#### 3.1.5. FLNC

*FLNC* encodes filamin C protein that localizes in Z-disc and maintains the integrity of the sarcomere by crosslinking actin filaments at the sarcomeric Z-disc. It has been recently recognized as a gene associated with isolated cardiomyopathies. Truncating variants in *FLNC* are strongly enriched in an overlapping phenotype of DCM and left-dominant arrhythmogenic cardiomyopathy (57), as *FLNC* is highly intolerant of loss-of-function variants. Missense variants in *FLNC* have been associated with autosomal-dominant restrictive cardiomyopathy (MIM #617047) (58). HCM also seems to be mainly associated with missense variants, which cause changes in the secondary protein structure (MIM #617047). Only 13 missense variants have been supported to be pathogenic by functional and/or segregational studies (59). Over the past year, the first familial HCM caused by a splicing mutation in *FLNC* was reported (60). Later, strong evidence for the involvement of *FLNC* in HCM was confirmed: a novel missense variant Ile1937Asn with complete penetrance and poor outcomes has been identified in a large 3-generation French-Canadian family with excellent segregation data (61). Interestingly, most missense variants and the aforementioned splice variant are located in the ROD2 domain of *FLNC* gene, which is also participating in cell signaling, and can be considered as a mutational hotspot region for HCM-related *FLNC* variants.

#### 3.1.6. JPH2

Junctophilin 2, encoded by *JPH2*, is a major isoform of its family in the heart that localizes in sarcoplasmic reticulum within junctional membrane complexes. It serves to bridge plasma membrane and sarcolemma, interacts with L-type Ca2+ channels, caveolin and RyR2 to regulate Ca2+ induced Ca2+ release. In 2023 a comprehensive systematic review of the cardiac manifestation of all *JPH2* pathogenic variants was published (HCM, MIM #613873 and DCM, MIM #619492) (62). Patients with autosomal dominant heterozygous variants developed HCM (76%) and arrhythmia/SCD (24%). Patients with homozygous/compound heterozygous loss-of-function variants developed DCM and early-onset heart failure. Among a total of 61 variant-positive individuals, 47% had HCM. However, additional studies are still needed to provide conclusive evidence for this association.

#### 3.1.7. KLHL24

*KLHL24* encodes the Kelch-like protein 24 that is commonly expressed in the skin and heart and involved in intercellular compliance networks. Kelch-like protein 24 regulates ubiquitination and subsequent proteasome degradation of keratins in keratinocytes and desmin in cardiomyocytes; the dysregulation of this process can result in both an excessive degradation and, on the contrary, an accumulation of intermediate filament proteins (63). Pathogenic variants in *KLHL24* gene may cause solitary skin (MIM #617294) or heart disorders (HCM, MIM #620236) (64), as well as combined so-called cardiocutaneous syndromes (MIM #617294). Causal variants in *KLHL24* are rare in the general population, and establishing their pathogenicity is challenging. A summary of the functional impact of eight *KLHL24* variants on protein function has recently been published (63). Generally, patients with heterozygous gain-of-function variants can develop DCM phenotype with desmin deficiency, meanwhile, HCM with desmin-overload has been determined in patients with homozygous loss-of-function variants. Parents of HCM patients carrying the heterozygous loss-of-function variants did not display signs of cardiac disease (65).

#### 3.1.8. PLN

The *PLN* gene comprises only one exon and encodes a short protein phospholamban consisting of 52 amino acids, which is a key regulator of cardiac contractility: it inhibits cardiac muscle sarcoplasmic reticulum Ca(2+)-ATPase, thereby mediating contraction and relaxation. Due to its size, the number of possible causal variants is limited. However, mutations in *PLN* are associated with different cardiomyopathies (HCM, MIM #613874; DCM, MIM #609909, and arrhythmogenic cardiomyopathy). Thereby, *PLN* was classified as a definitively associated gene with an “intrinsic cardiomyopathy”. Among all *PLN* variants, only p.Leu39Ter has been shown to be significantly enriched in HCM (66, 67).

#### 3.1.9. SVIL

*SVIL* encodes supervillin, which is a large multi-domain actin and myosin-binding protein, localized in Z-disc. Its muscle isoform plays a role in myofibril assembly. In 2020 the homozygous loss-of-function variants in *SVIL* were described in individuals with skeletal myopathy and slightly hypertrophic LV walls (MIM #619040) (68). *SVIL* gene has been just recently associated with HCM; a 10.5-fold excess burden of rare truncating *SVIL* variants in HCM cases has been demonstrated in the largest GWAS (30). In one family, *SVIL* variant p.Gln255* was carried by two affected cousins. However, more co-segregation evidence is still required for supporting variants’ pathogenicity.

#### 3.1.10. TRIM63

To maintain normal cardiac function, sarcomeric proteins undergo constant turnover by UPS. Ubiquitin ligases direct the addition of ubiquitin to target proteins, marking them for degradation (69). One of the ubiquitin ligases is muscle-specific RING-finger protein 1, encoded by the *TRIM63* gene. The protein localizes to the Z-disc and M-band of the sarcomere, where it interacts with myosin heavy chain, troponins, tropomyosin, and titin. Salazar-Mendiguchía et al. demonstrated an association between homozygous and compound heterozygous rare variants in *TRIM63* and the development of HCM. The family evaluation confirmed a recessive pattern of disease inheritance, as heterozygous carriers were healthy (70). This gene is responsible for approximately 0.4% of HCM cases, but an increasing number of *TRIM63*-associated HCM cases is expected (71).

*Due to the small number of cases, obtaining strong evidence for the causative role of variants in non-sarcomeric genes in HCM is a challenge. Including candidate genes in routine genetic panels will push on enough data to elucidate this. As segregational data for rare variants are limited, functional investigations appear to be more productive in uncovering the impact of non-sarcomeric genes in HCM. Since more and more non-sarcomeric genes are recognized to be disease-causing, the list of candidate genes must be reassessed regularly*.

### 3.2. Genes of HCM phenocopies

Phenocopies of HCM are named the diseases that have heart changes similar to HCM. The vast majority of these rare diseases are systemic and inherited. Some patients have very subtle extracardiac features of a syndromic disorder and the real origin of HCM phenotype may be overlooked by medical professionals. The patients are followed for years with presumed sarcomeric HCM until genetic testing is done. The main genes that are classified as having a definitive association with their respective syndromes and may cause isolated, or seemingly isolated, LV hypertrophy (20), are presented in Table 2.

**Table 2:** Definitive syndromic genes and their variants that may cause cardiac (HCM) phenotype.

Gene (main transcript)	Main phenotype MIM identifiers (other cardiac signs)	Disorder by pathogenesis	Variants (Reference)
*CACNA1C* (NM_000719.7)	Timothy syndrome MIM #601005 (CHD, LQTS, AF, SSS)	Long QT syndrome	c.1552C>T (p.Arg518Cys) c.1552C>T (p.Arg518His) (72, 73)
*DES* (NM_001927.4)	Desminopathy MIM # 601419 (AV-block)	Neuromuscular disorder	c.1216C>T (p.Arg406Trp) (74, 75)
*FHL1* (NM_001159699.2)	Emery-Dreifuss MD MIM #300696 (atrial standstill, AV-block)	Muscular dystrophy	c.673T>C (p.Cys225Arg) (76) c.134delA (p.Lys45Serfs), c.827G>C (p.Cys276Ser) (77) c.599_600insT (p.Phe200fs32X) (78)
*GLA* (NM_000169.3)	Fabry disease MIM #301500 (short PR, AV-block)	Storage disease	c.640–801G>A (IVS4+919G>A) (79) c.644A>G (p.Asn215Ser), c.888G>A (p.Met296Ile), c.902G>A (Arg301Gln), c.982G>C (p.Gly328Arg) (80); c.427G>C (p.Ala143Pro) c.758T>C (p.Ile253Thr) c.613C>A (p.Pro205Thr) c.386T>C (p.Leu129Pro) c.1072G>A (p.Glu358Lys) (81) c.337T>C (p.Phe113Leu) (82)
*LAMP2* (NM_002294.3)	Danon disease MIM #300257 (WPW, short PR, AV-block, AF, high QRS voltage)	Storage disease	Any in females (83)
*PRKAG2* (NM_016203.4)	PRKAG2 cardiomyopathy MIM #600858 (bradycardia, CCD, WPW, short PR, AF, PSVT)	Storage disease	All (84)
*PTPN11* (NM_002834.5)	Noonan syndrome MIM #163950 (CHD)	RASopathy	c.922A>G (p.Asn308Asp) (81) c.1528C>G (p.Gln510Glu) (85) c.1403C>T (p.Thr468Met) (86)
*RAF1* (NM_002880.4)	Noonan syndrome MIM #611553 (CHD)	RASopathy	c.769T>C(p.Ser257Pro) (86) c.779C>T (p.Thr260Ile) (87)
*RIT1* (NM_006912.6)	Noonan syndrome MIM #615355 (CHD)	RASopathy	c.170C>G (p.Ala57Gly) (88)
*TTR* (NM_000371.4)	Transthyretin amyloidosis MIM #105210 (low or “normal” QRS voltage, AV-block)	Infiltrative disease	c.424G>A (p.Val142Ile) c.391C>A (p.Leu131Met) c.238A>G (p.Thr80Ala) c.262A>T (p.Ile88Leu) c.323A>G (p.His108Arg) c.118G>A (p.Val40Ile) (89)

CHD, congenital heart disease; LQTS, long QT syndrome; AF, atrial fibrillation; SSS, sick sinus syndrome; MD, muscular dystrophy; WPW, Wolff Parkinson White; CCD, cardiac conduction defects; PSVT, paroxysmal supraventricular tachycardia.

Aside from P/LP variants, there are a growing number of reported VUS in these genes. In some cases, they can play a cumulative role in phenotype formation along with major sarcomeric mutations (90). Regarding the genes of storage diseases, there is still a big question about which variants lead to significantly reduced enzyme activity that may result in disease (82). It is especially important to be sure in diagnosis based on genetic tests in late-onset cases when symptoms of inherited and common diseases (LV hypertrophy, stroke, renal failure) are overlapping. For example, the determination of the pathogenicity of *GLA* variants (Fabry disease, MIM #301500) is crucial to start the specific enzyme replacement therapy (ERT) and must be accompanied by measurement of sphingolipid concentrations (82).

*As the extracardiac features of rare diseases can be overlooked by clinicians, defining a precise cause of LV hypertrophy may facilitate clinical re-assessment and correct diagnosis. Keeping in mind that some HCM phenocopies are treated with targeted therapies (for example, ERT in Fabry disease or TTR stabilizers in transthyretin amyloid cardiomyopathy) sequencing of syndromic genes within HCM panel has a high clinical benefit*.

## 4. Searching for missing HCM heritability

Since over half genotyped patients have negative genetic test results the deciphering etiology of HCM is ongoing. There are two approaches to explain genotype/sarcomere negative HCM: (1) new undiscovered genes contributing to the Mendelian form of the disease, and (2) the oligogenic/polygenic nature of HCM.

### 4.1. How to find new candidate genes?

GWAS using NGS-based genotyping technologies in large cohorts of patients with cardiovascular diseases and controls made it possible to find new genome loci associated with a disease or with disease LV traits (91). Follow-up bioinformatic analyses of these loci identified candidate genes that are enriched in cardiac functions including myocardial growth and sarcomere organization (10, 14, 31). Another way of searching for novel disease-associated genes is to investigate the underrepresented populations, such as those of the Middle East, where a proportion of consanguineous families and recessive type of transmission is high. In these populations, the prevalence of variants in “minor” genes is increased due to recessive inheritance patterns and the presence of founder variants. Bi-allelic variants are often associated with an earlier and more severe clinical presentation of HCM that gives the opportunity of uncovering novel disease-causative genes if use whole exome sequencing (WES) or whole genome sequencing (WGS) as a first-line test (92, 93). Finally, using the extended panels comprising multiple candidate genes in genotype-negative patients with other affected family members available for segregation analysis can help to discover novel genes (46, 55, 70). Important to note, that a significant proportion of findings in candidate genes cannot be used for predictive family screening because of the lack of evidence for the involvement of these genes in the pathogenesis of the disease. Nevertheless, the genetic finding may indicate the direction of movement in searching for the molecular cause of the disease in each patient, and the accumulation of even clinically unsuitable information from thousands of patients can accelerate the deciphering of sarcomere-negative HCM pathogenesis.

### 4.2. WES and WGS in routine practice

WGS is known to analyze 90% of the genome and all exons, offering the potential to identify disease-causing CNVs and structural variations (deletions, insertions, duplications, inversions, and translocations), repeat expansions, and splicing and regulatory variants (94). On the contrary, WES examines exons only (1%–2% of the genome), which disables the detection of non-coding and structural variants. Nevertheless, currently, the diagnostic rate of WGS did not differ significantly from that of WES possibly due to the much broader use of WES over WGS and/or to the substantial cost difference between the two (95). However, it has become evident that WGS is capable of achieving molecular diagnoses for cases undiagnosed by WES and especially in genotype-negative HCM patients who have not been diagnosed using standard panels (95–98). In a recent meta-analysis, the power of WES and WGS in influencing clinical management ranged between 2% and 100%, and a much higher pooled clinical utility of both WES and WGS (increasing 2.6% each year as illustrated by the meta-regression) compared with previous evidence was illustrated (95). The creation of sufficiently large genome-wide datasets will allow us to identify genes/variants with moderate size effects and validate PRS (99).

Currently, all familial HCM cases considered genotype-negative or sporadic genotype-negative cases with overt severe phenotype are the target group for this type of genetic study. The yield of WGS as a second-line test in appropriate HCM patients is up to 20% and about half of the findings belong to protein non-coding regions of the genome (100). However, the use of WES/WGS in HCM clinical practice might be cost-effective. There must be a certainty that all definitive and moderate validity HCM-associated genes have been analyzed using cheaper methods, the quality of sequencing (coverage) was satisfactory, and the results were interpreted by a trained multidisciplinary team. The price of WES/WGS is decreasing as a consequence of cheapening of NGS (101), and in the near-term these methods may become first-line tests in a diverse range of patient groups enable laboratories to benefit from increased standardization, re-analyzing of genotype-negative cases over time, and implement gene-discovery approach (99). Accelerating WGS/WES data will generate meaningful cost-effectiveness estimates, providing empirical evidence to inform clinical management and allocate healthcare resources at a national level.

The limitation of WES, working through capturing and sequencing all protein-coding regions of the genome, lies in the suboptimal coverage of some genes, owing to difficulties in the design of probes. Among the limitations of routine usage of WGS, there is the need for computational infrastructures suited to store and analyze terabytes of data (99). It should be noted that there is still a great lack of diversity in genomic research that manifests in the underrepresentation of populations other than White individuals; it limits the usefulness of WES and WGS, complicating the interpretation of genetic testing results. But the most challenging in a WES/WGS utility is a potential wrong false positive interpretation of a large number of variants, thus certain expertise in variant interpretation is crucial.

### 4.3. Non-coding regions of genome

Non-coding regions account for about 97% of the human genome. A vast majority of disease-associated variants (90%) that have been identified by GWAS reside within protein non-coding regions (102) and may be involved in regulating the expression of protein-coding genes and thereby contribute to the clinical manifestation of the diseases. By today, there is sufficient evidence that variants within promoter, enhancer, untranslated, splice, and intronic regions support a strong association with cardiomyopathies, including HCM [reviewed in (103)]. Furthermore, according to ClinVar, a significant percentage of non-coding variants in splice sites (−60%) and UTRs (−5%), are classified as P or LP (www.ncbi.nlm.nih.gov/clinvar). Advanced sequencing- and imaging-based technologies together with powerful computational methods enable us to improve the understanding of three-dimensional (3D) genome architecture and uncover the mechanism of non-coding variants affecting coding genes. There are plenty of examples where using chromosome conformation capture (3C)-based technologies successfully links non-coding variants to their target genes and prioritizes relevant tissues or cell types (104). The other methodology of searching for non-coding rare variants in association with diseases is under development (105).

#### 4.3.1. Deep intronic variants

Most data about the association between non-coding variants and HCM are related to deep intronic variants. These variants are located sometimes more than 100 base pairs away from canonical exon-intron sites and are not covered by conventional NGS or WES restricted to exons and exon-intron boundaries. Deep intronic variants most commonly lead to intron sequence (pseudo-exon) inclusion in the mature messenger RNA due to the activation of non-canonical splice sites or changes in splicing regulatory elements. Additionally, these mutations can disrupt transcription regulatory motifs and inactivate non-coding RNA genes (106), which are often hidden within introns of protein-coding genes and involved in the regulation of gene expression. The longer a gene the more likely it is to be affected by deep intronic variants. It is confirmed by the fact that numerous deep intronic mutations have been described in particularly long genes such as those associated with neurofibromatosis (107) and Duchenne muscular dystrophy accompanied by X-linked DCM (108–110).

In cohorts of HCM patients, deep intronic variants have been detected mainly in *MYBPC3* gene. Deep intronic *MYBPC3* variants are presented in 1% of all genotyped HCM cases and in 2.2%–9% of genotype-negative patients who do not carry pathogenic coding or canonical splice variants in HCM-associated genes (100, 111, 112). The patients with deep intronic causative variants have the classic signs of HCM as the other patients carrying mutations in canonical regions (112). Moreover, *MYBPC3* c.1224-52G>A is one of the most common variants among all known HCM variants currently associated with HCM (detected in about 1% of HCM probands) (113), and second only to the *MYBPC3* c.1504C>T (p.Arg502Trp) variant, the most common *P* variant in the general HCM population (detected in 1.7% of HCM cases) (113). *MYBPC3* c.1224-52G>A results in the inclusion of 50 intronic nucleotides in the transcript which is predicted to lead to a frameshift in the amino acid sequence and the insertion of a premature stop codon. It is noteworthy that the role of *MYBPC3* c.1224-52G>A in HCM was discovered by another intronic variant *MYBPC3*Δ25 (a 25 base pair deletion within intron 32), which is too common to be a *P* [although it was associated with South Asian HCM for a decade (114)], but proved to be a marker of the haplotype bearing both the common *MYBPC3*Δ25 variant and a rare apparently important HCM variant *MYBPC3* c.1224-52G>A (113). Another example of a common deep intronic variant associated with HCM phenotype is c.639 + 919 G>A located in a *GLA* gene. This variant causes cryptic splicing, markedly reducing the amount of wild-type *GLA* mRNA and the development of late-onset cardiac Fabry disease—a phenocopy of HCM (79, 115). It was found to be the most prevalent *GLA* mutation in the Taiwanese newborn population (82% of all findings in *GLA* gene) (79) with a frequency 1 in 875 males (116). Further investigations of this particular variant demonstrated the therapeutic feasibility of splicing correction with specific splice-switching oligonucleotides (117) that may become an alternative treatment for Fabry disease which is currently treated with ERT. The deep intronic variants associated with HCM have been reported in some other genes—*VCL*, *PRKAG2,* and *TTN* (32). Analysis of one family suggested that *VCL* c.499 + 367T>C variant is not sufficient to cause disease development alone but could have a modifier effect on coexisting sarcomeric *MYBPC3* splice site variant c.1227-13G>A*.* The carriers of *MYBPC3* c.1227-13G>A alone do not manifest the disease, while family members that were *MYBPC3-VCL* double heterozygous were clinically affected (32). Besides WGS, the whole sequencing of a certain gene can be used to search deep intronic variants (32). Target NGS sequencing of intronic regions of *MYBPC3* gene led to molecular diagnosis in a significant proportion (6.5%) of the cohort of initially genotype-negative HCM patients (111).

Assessing the pathogenicity of deep intronic variants is very challenging. Analysis of mRNAs in affected tissues of patients is crucial for confirming the pathogenicity of deep intronic variants especially because such variants may be amenable to correction with antisense oligonucleotide therapies (106). However, biopsy samples are not available in most HCM patients, functional studies are beyond the scope of most clinical laboratories, and *in silico* splicing prediction tools are imperfect. These factors create barriers to the addition of deep intronic variants onto genetic panels.

*Thus, sequencing of at least deep MYBPC3 intronic regions should be done routinely. The deep intronic sequences of other reported genes can be analyzed secondarily. The validation functional analysis should be done in cases where heart biopsy is available*.

### 4.4. CNVs

The overwhelming majority of genetic variants (>95%) associated with cardiomyopathies are single substitutions of one nucleotide for another or small insertions/deletions of less than 20 nucleotides. However, some genes and even whole genome regions are prone to have large deletions or duplications that are called CNVs and may also cause inherited diseases. Detection of this type of variants requires applying specific technical and/or computational methods (118–122). Small series of studies have evaluated CNVs in HCM. CNVs were reported predominantly for sarcomeric genes: *MYBPC3* (119–121, 123–127), *MYH7* (121, 124, 125), *TNNT2, TNNI3*, and *ACTC1* (119). Among them, there were only two recurrent CNVs. The partial tandem of duplication of *MYH7* and *MYH6* which is predicted to create a hybrid gene causing HCM by incorporation of a “poison peptide” in the sarcomere has been reported twice; in one family it was shown to segregate with HCM in seven relatives (121, 124). Another recurrent CNV is a 3505-bp deletion that encompasses the exons 28–35 of *MYBPC3* gene (126, 127). CNVs in non-sarcomeric genes such as *PDLIM3*, *LMNA* (119), *MYOZ2* (85), and *PLN* (120) were also suspected to be a cause of HCM but additional evidence is still required. The possible causal role of duplications is more difficult to judge than deletions. Although the proportion of HCM cases caused by CNVs is small (<1%) (85, 119, 121), for the subset of patients with clearly interpretable CNVs these findings have direct clinical implications. It should be noted that even in the presence of P/LP single nucleotide variant, the CNV might be found and be clinically relevant, especially in early disease onset and severe clinical course cases.

*Therefore, the techniques that allow CNVs to be found should be applied systematically in gene panels for HCM. Sarcomeric genes, especially MYBPC3, are the front-line candidates for searching CNVs in HCM patients*.

### 4.5. VUS

A large number of genetic variants are represented by VUSs. In general, VUSs are absent or have a low frequency in the general population (minor allele frequency, MAF < 0.1), have not been previously described in affected individuals, or there is no data to prove cosegregation in families, or the affected genes have little evidence of involving in pathogenesis (often related to minor and candidate genes), or their functional mechanism differs from that expected and is likely not to translate into protein alteration (16). Furthermore, VUSs are differently evaluated by clinicians and researchers, and different laboratories do not necessarily adopt the same standardized reporting format (128). Such a severe heterogeneity in VUS analysis creates a knowledge gap that makes VUSs problematic to use, overlooking potentially disease-relevant information; it also requires consensus from laboratories, clinicians, genetic counselors, patients, and policymakers to avoid ethical issues for better practices. At the same time, reporting VUS may cause confusion and lead to unnecessary medical action or even serious clinical consequences in terms of overdiagnosed inherited conditions with a high risk of SCD requiring implantable cardioverter-defibrillators, lifelong follow-up and psychological burden towards offspring (129, 130) as well as incorrect genetic cascade screening of the family. Thus, determining the causative role of VUS in a patient's phenotype is the most challenging part of genetic testing.

VUSs in sarcomeric genes are identified in about 10% of HCM patients and this subgroup demonstrates intermediate outcome risk between sarcomere-positive and sarcomere-negative ones (36). It means that a significant proportion of sarcomeric VUSs are involved in disease development. However, most of VUSs (85%) are detected in uncommon genes (131) which information is extremely scarce and the risk of overestimating the association between the proposed variant and the disease with harmful consequences for the family is high.

Over the past decade, great efforts have been made by international collaborators to improve and facilitate the primary variant interpretation process (132). Important resources have been developed. Genome Aggregation Database (gnomAD) is the largest public open-access human population genome dataset to date which helps to identify variants that are too common to be causing a patient's disease (gnomAD v3.1.2 https://gnomad.broadinstitute.org). Being enormously big, this database provides additional tools for correct variant interpretation: careful annotation and curation of loss-of-function variants (not all of them actually result in a loss of protein function and are pathogenic) and the degree of intolerance of genes to this type of variants (for example, *MYH7* gene is tolerant to loss-of-function variants, thus, only missense *MYH7* variants are pathogenic); the difference in exons expression on tissue-level can help to check whether the exon containing a variant of interest is expressed in the damaged organ; updated information on the involvement of non-coding region variants in human diseases; etc. ClinVar is another open database aggregating interpretations of the clinical significance of variants from different sources (http://www.ncbi.nlm.nih.gov/clinvar/). Since a rarity of a variant in the population is recognized as the most important criterion for pathogenicity (but is insufficient alone), improved analytical approaches including statistical models based on population frequency thresholds are being developed to determine which genes and variant classes are interpretable for the Mendelian form of the disease (21, 34, 133, 134). In 2018 the ClinGen Inherited Cardiomyopathy Expert Panel (CMP-EP), founded to regularly assess the veracity of all gene-disease claimed associations, developed the adapted version of ACMG/AMP framework for *MYH7*-associated cardiomyopathies with the expectation that most rules would apply to the other cardiomyopathies-associated genes. The CMP-EP adaptation affected MAF thresholds, the use of segregation data, and a semi-quantitative approach to counting multiple independent variant occurrences where fully controlled case-control studies are lacking (135).

To resolve the status of a proposed VUS, in an ideal scenario, it is necessary to prove its segregation with the disease in a large family (≥7 meioses) (135) as a cosegregation analysis within the family provides the most robust evidence of causality. In case the family is not available or the number of affected members is not statistically enough to follow for segregational analysis (majority of cases), we can periodically revise VUSs to assess if the variant should be moved to a pathogenic classification based on new evidence from the other families. From this point of view, publishing cases with even insufficient evidence is encouraged because the same or neighboring variants in larger families may be found by other researchers interested in exploring confirmatory functional data in collaboration. This can potentially contribute to clarifying the role of candidate genes (136). Large gene-centric case-control studies are another effective strategy to gather statistical evidence in support of association with disease for candidate genes when segregational data are not available. This approach has recently allowed proving the causative role of non-sarcomeric *FHOD3* (55) and *ALPK3* (46) genes in HCM development which now facilitates the interpretation of variants in these genes if they are found in patients with HCM phenotype. Another method to clarify the pathogenicity of VUSs is functional studies, such as RNA analysis for splice-site variants (137), which can detect their deleterious effect on the protein. This approach might be faster for a given family and should be applied more widely in appropriate cases. Of course, all of the above approaches to clarify VUS status are time-consuming and impose an additional financial burden on laboratories, insurance companies, or families, depending on the healthcare system.

*In an ideal scenario, presenting VUS in the genome of patients with clear phenotype in the absence of other definitive variants should be followed by recommendations to perform segregation analysis within the family if the pedigree is large and/or by a functional study to elucidate the pathogenicity of such variants. In case this is unfeasible, these data must be re-evaluation over time. If VUS(s) accompanies the definitive disease-causative variant the modifier effect or inheritance of multiple variants cannot be ruled out and must be verified within the family. Today this scheme can be applied in isolated cases first of all due to cost consumption*.

### 4.6. Autosomal-recessive and X-linked HCM

Despite HCM being predominantly an autosomal dominant disease, some HCM cases with recessive and X-linked patterns of inheritance have also been described. As previously mentioned, exclusively an autosomal-recessive inheritance of HCM has been demonstrated for *TRIM63* (70, 71) and *KLHL24* (64) genes. A variant-specific inheritance of HCM has been also described: some mutations in *MYL2* and *MYL3* genes cause an autosomal dominant, while the others—an autosomal recessive pattern of inheritance of the disease (138, 139). Three genes associated with HCM phenocopies (*FHL1*, *GLA,* and *LAMP2*) are X-linked: male carriers of mutations develop overt disease phenotype earlier while heterozygous females are mild / not affected or have a late-onset presentation (77–83).

*Thus, the interpretation of variants in these particular genes, as well as new candidate genes and novel variants, must be analyzed in accordance with alternative patterns of inheritance*.

### 4.7. Polygenic HCM

It is now believed that some HCM patients have a non-Mendelian form of the disease. Some common variants with MAF > 0.01 can exhibit disease-causing effects if they are present together in the genome (10, 140, 141). Large-scale GWAS demonstrated that common genetic variants, located in many loci, contribute substantially to HCM risk, and highlighted the complex genetic architecture of HCM (10, 14, 30, 142). The more variants in the genome the higher PRS for HCM development. It is assumed that a high PRS also influences variability in penetrance and expressivity of Mendelian rare pathogenic variants clarifying previously unexplained clinical heterogeneity of monogenic HCM (10, 14, 30). But a strong polygenic inheritance was noted particularly for sarcomere-negative patients (10). There is also a group of low-frequency variants of small or intermediate effect size that cannot result in the disease themself but together with other rare variants or PRS can express the phenotype (91). Thus, in some cases we can talk about oligogenic inheritance of HCM.

To date, there are several lists of polymorphic variants located in the coding genome that can predict HCM development and adverse outcomes in both carriers of sarcomeric variants and sarcomere-negative HCM patients (10, 14, 143). Amongst sarcomere-positive carriers in the general population, HCM penetrance differs 10-fold between those in the highest and lowest PRS quintiles (143). Notably, some risk variants are located in genes already established for Mendelian HCM, such as *ALPK3*, *FLNC*, *PLN*, and others (10, 14), which should be taken into account when interpreting findings. Using GWAS-derived PRSs seems promising in risk stratification and makes clinical interventions such as implantable cardioverter-defibrillator more targeted and family screening more productive.

*After validation of the utility of PRS in clinical trials and establishing the thresholds for different cohorts (ethnicity, general population* vs. *family members) and purposes (diagnostic, prognosis, selection for specific treatment, and therapeutic response), the PRS could be calculated in each HCM patient regardless of the presence or absence of pathogenic rare variants*.

## 5. MiRNAs

MicroRNAs (miRNAs) are small non-coding RNAs that form a coordinated regulatory system and control the regulation of a variety of genes involved in fundamental biological processes, such as tissue differentiation, proliferation, apoptosis, stress response, etc. (144). The sequence-specific regulation of target gene expression by miRNAs is one of the important mechanisms of the realization of genetic information, which can make a significant contribution to the penetrance of genetic variants associated with HCM and thus in the general heterogeneity of disease phenotype. Moreover, genetic variants in the miRNA binding sites located in 3′-untranslated regions of HCM-associated genes can change the binding ability of miRNAs, which leads to miRNA reorienting to other targets and therefore causing changes in cellular processes. Thus, changes in miRNA levels in the cell and potentially in circulating fluids may indicate the carriage of a P/LP variant in a HCM patient. It is also known that there are several miRNA genes located in the introns of sarcomeric HCM-associated genes (145); variants in splice sites of the host gene for intronic miRNAs or of the clustered miRNAs could result in aberrant expression patterns as well.

Many studies have already been conducted on the role of miRNAs in HCM, and there is a great deal of evidence that miRNA levels in different biological materials are correlated with the disease phenotype and prognosis [reviewed in (146)]. Future miRNA studies may focus on pinpointing the individual miRNAs that are foremost associated with a particular HCM trait, or to compile miRNA panels that can be used depending on the clinical task, such as individual assessment of the phenotype prediction, complications development, and its type.

Currently, the use of a genotype-based approach to identify biomarkers of HCM and its progression seems extremely relevant. Recently, by using such an approach we demonstrated that circulating miR-499a-5p identifies with high sensitivity and specificity HCM patients with P/LP variants in the *MYH7* gene (147). Extending this approach for detecting patients with P/LP variants in the *MYBPC3* or other HCM-associated genes could improve the diagnostic accuracy and efficacy of genetic tests, pinpointed patients tested negative for P/LP variants, and cut the cost of subsequent genetic analysis in some patients by reducing the number of genes to be sequenced.

Another study showed the possibility of using miRNAs to identify pathology in apparently healthy subclinical sarcomeric variant carriers (148). Despite normal electrocardiogram and echocardiogram in P/LP sarcomeric variant carriers miR-26b-5p, miR-301a-p, and miR-31-5p strongly discriminated subclinical HCM and healthy controls, suggesting that sarcomeric variants are resulting in subtle changes that cannot be detected by standard imaging-based methods. More importantly, miR-181a-5p, miR-181c-5p, miR-328-3p, miR-301a-3p, miR-193b-3p, miR-142-3p were found to be differentially circulated in subclinical HCM patients with early phenotypic manifestations and those without early phenotypic manifestations, suggesting that although LV hypertrophy is not yet evident, a biological effect from the sarcomeric variant is present based on electrocardiogram changes, impaired LV relaxation, and alterations in circulating miRNA patterns. Interestingly, although miRNAs could discriminate clinically over HCM from subclinical HCM without early phenotypic manifestations with high sensitivity and specificity (top three miRNA: miRNA-193b-3p, miRNA301a-3p, and miRNA-181a-5p), circulating miRNAs failed to discriminate clinical HCM and subclinical HCM with early phenotypic manifestations, suggesting biologic commonality between these two states. The authors conclude that the presence of early phenotypic manifestations and a shifting of the miRNA profile may portend a rapid transition to clinically overt HCM.

*Combining miRNA and genetic analyses will provide more personalized clinical treatment, targeting people who may benefit most from more aggressive treatment, rather than those who may have delayed or absent penetrance. MiRNAs seem to act both as markers for risk stratification and targets for personalized treatment in HCM patients*.

## 6. Discussion

The current knowledge about HCM dictates steps forward in genetic investigations of this disease. The genetic test for one patient may/should include the analysis of (1) sarcomeric genes; (2) genes of HCM phenocopies; (3) validated HCM non-sarcomeric genes; (4) non-coding variants and CNVs in at least sarcomeric genes; (5) common risk variants with PRS calculation; (6) VUS pathogenicity by functional and co-segregational studies in families; (7) miRNAs with risk score assessment. The list of candidate genes must be updated regularly; there should be particular attention to the pattern of disease inheritance and the presence of multiple rare variants in some clinical cases.

Applying the above approach to further studies on HCM leads to the rapid accumulation of a huge amount of complex data requiring the usage of artificial intelligence to analyze. Deciphering of genotype-phenotype correlations for new regions will contribute to a better understanding of the molecular mechanisms of the disease in each patient which is required for early diagnosis, prognosis, and personalized treatment including preventing the development of the disease by rapidly developing genetic technologies. This looks especially appealing in the context of recent breakthrough studies that shed more light on the therapeutic potential of genome editing strategies in HCM treatment.

Currently, there are several fastest-growing tools for the manipulation of DNA to correct cardiac disorders: CRISPR-Cas9 editing, base editing (BE), and prime editing (PE). CRISPR is a two-component system consisting of guide RNA and a Cas9 nuclease. The Cas9 nuclease cuts the DNA within the region defined by the guide RNA. BE is the newest method of gene editing derived from CRISPR-Cas9. This technology uses a “catalytically dead” Cas9 that cannot cleave DNA and a DNA deaminase domain catalyzing the deamination of either adenosine or cytidine resulting in base conversions to guanine or thymine, respectively (149). Compared to BEs, PE enables all 12 possible base-to-base conversions, as well as insertions and deletions, without requiring double-stranded breaks or donor DNA (150). PE system consists of a nickase Cas9 conjugated with an engineered reverse transcriptase paired with a prime-editing guide RNA that both specifies the target site and encodes the desired edit. Therefore, state-of-the art genome editing technologies, with their simplicity and precision, hold great promise for the correction of point mutations in human genetic diseases, including HCM. Two independent studies focusing on the *in vivo* editing of the pathogenic HCM-associated variant c.1208G>A (p.R403Q) located in *MYH7* gene were recently published (151, 152). One study identified an adenine BE and single-guide RNA system that efficiently corrected human c.1208G>A pathogenic variant with minimal bystander editing and off-target editing at selected sites (151). The delivery of BE components attenuates pathological manifestations of HCM in patient-derived induced pluripotent stem cell cardiomyocytes and a humanized HCM mouse model. In another study, two different genetic therapies—an adenine BE and a potent Cas9 nuclease delivered by AAV9—were evaluated to prevent HCM in mice carrying the heterozygous c.1208G>A pathogenic variant (152). Applying RNA-guided adenine BE corrected the pathogenic variant in ≥70% of ventricular cardiomyocytes and maintained durable, normal cardiac structure and function. An additional dose provided more editing in the atria but also increased bystander editing. RNA-guided Cas9 nuclease effectively inactivated the pathogenic allele; however, due to the observed dose-dependent toxicity, a narrow therapeutic window is required to maintain health. These findings demonstrate considerable potential for single-dose genetic therapies to correct or silence pathogenic variants and prevent the development of HCM. Other gene editing innovations and their applications in the treatment of cardiomyopathies are summarized in a recently published review (153).

It should be noted that there is no consensus on the use of an advanced genetic panel for HCM in clinical practice (33, 34, 100). Although promising, a range of barriers impedes the above-mentioned techniques from being implemented in cardiology practice. These barriers include economic concerns (e.g., perceived increased cost of broadened genetic testing precluding insurance coverage), concerns related to knowledge, attitudes, and practices on clinicians’ part, and psychological distress and potential negative impact on self-perception on patients’ part, as well as ethical concerns related to increased stigma and discrimination. Ongoing reductions in the costs of DNA sequencing, and improvements in variant analysis will support the cost-effectiveness of such an approach. A variety of recommendations can be also followed to overcome such barriers to a successful implementation of broadened genetic testing in real practice tomorrow.
